# Dr. Harris A. Bostwick

**Published:** 1913-01

**Authors:** 


Dr. Harris A. Bostwick, retired, died at his home in Warsaw, Dec. 6. He had practiced in Rochester, Hermitage and Silver Springs.

